# Supporting Hospital Doctors in the Middle East by Email Telemedicine: Something the Industrialized World Can Do to Help

**DOI:** 10.2196/jmir.9.4.e30

**Published:** 2007-10-22

**Authors:** Victor Patterson, Pat Swinfen, Roger Swinfen, Emil Azzo, Husen Taha, Richard Wootton

**Affiliations:** ^5^Centre for Online HealthUniversity of QueenslandBrisbaneAustralia; ^4^Department of SurgeryEmergency HospitalErbilIraq; ^3^Department of ObstetricsAzadi General HospitalKirkukIraq; ^2^Swinfen Charitable TrustCanterburyUK; ^1^Neurology DepartmentRoyal Victoria HospitalBelfastUK

**Keywords:** Telemedicine, electronic mail, developing countries, Middle East

## Abstract

**Background:**

Since 1999, the Swinfen Charitable Trust has operated an email referral system between doctors in the developing world and specialists in the industrialized world. Since 2001, it has expanded its operation into the Middle East, in particular Iraq, an area of considerable conflict.

**Objectives:**

The aim was to compare referral patterns to the Trust from the Middle East with those received from the rest of the developing world and to look for qualitative evidence of health gain.

**Methods:**

We analyzed referrals to the Swinfen Charitable Trust between July 2004 and June 2007 and compared these by speciality with those received from elsewhere during the same 3-year period. We asked two referring doctors for their views of the process, and we analyzed the total Middle Eastern referrals made to a single specialty (neurology).

**Results:**

Between July 2004 and June 2007, 283 referrals were received from four countries in the Middle East (Iraq, Afghanistan, Pakistan, Kuwait) and 500 cases were received from 22 other countries. The 283 cases resulted in 522 separate queries to specialists. The median time to specialist reply for the queries relating to the 283 Middle Eastern cases was 24.3 hours (interquartile range 6.1-63.3). There was a significant difference in case mix between the Middle East and the rest of the world (*P* < .001), with more obstetric referrals and fewer referrals in medical specialties and radiology. The referring doctors were helped greatly by the service. The neurologist was confident of the diagnosis in 20 of 26 referrals received (77%). Both referring doctors and the specialist were able to cite referred cases where management was improved as a result of the service.

**Conclusions:**

Email telemedicine can be used in areas of conflict such as the Middle East. Perhaps surprisingly, trauma referrals are not increased but obstetric referrals are. Supporting individual doctor-patient encounters in this way is therefore often beneficial and is easily expandable. As well as improving care for individuals, email telemedicine provides effective case-based learning for local doctors, leading to improved care for subsequent similar patients.

## Introduction

While there is no doubt that political stability and public health are immensely important in improving a nation’s health, patients become ill regardless and doctors are needed to treat them. The outcome of the doctor-patient interaction is crucially important to the patient concerned, and a number of factors will optimize it, one of which is the expertise and training of that doctor in the patient’s specific complaint. Provision of the highest standard of medical care is the objective of those who work in the medical profession in the Middle East, as elsewhere, and these underpinning values do not change in times of conflict. For this reason, doctors’ knowledge needs to be upgraded continuously during their medical career. This is difficult to achieve in many parts of the developing world. In Iraq, for example, 13 years of strict international sanctions and three devastating wars in the last 25 years have not just destroyed or depleted the infrastructure but also made it extremely difficult for doctors to keep up with developments in their specialties. Despite their best personal efforts, their knowledge may not be sufficient to meet the high standards of medical practice they set for themselves and, consequently, some doctor-patient outcomes will be adversely affected.

**Figure 1:**
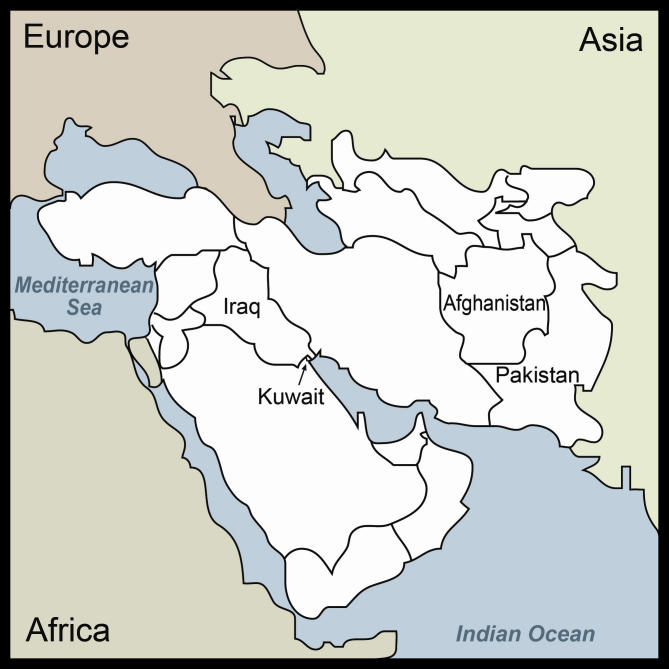
Middle Eastern countries with email telemedicine sites

An email telemedicine system was set up in 1999 by the Swinfen Charitable Trust in order to try and improve this situation. This has been described elsewhere [1] but is essentially simple in concept: hospital doctors in the developing world send an email message about a difficult patient that is passed on to a specialist in the industrialized world who replies with advice. In the last few years, links have been established with Iraq (29 sites), Pakistan (6), Afghanistan (3), and Kuwait (1) (Figure 1). We describe below how this system has worked in the Middle East, how referral patterns there differ from other parts of the developing world, and how both referring doctors and specialists feel about it.

## Methods

### The Referral Process

After hospitals wishing to participate have identified themselves to the Swinfen Charitable Trust, equipment, such as a digital camera and tripod, is provided and appropriate training is arranged. Referrals are sent by email to the single email address of an automated message-handling server (AutoRouter) [2] with anonymized clinical photographs or images as attachments. One of the system operators allocates the message to a relevant specialist who then replies to the AutoRouter, which forwards the reply to the referring doctor. The AutoRouter handles any further email dialogue automatically and alerts the system operators if an initial reply is not received within 48 hours. If a reply is not received within 2-3 days, the case is allocated to a different specialist. Specialists are hospital consultants throughout the world who give their time freely and endeavour to reply as soon as possible. The network started with a single hospital in Bangladesh and now serves 118 hospitals in 29 countries that are connected to 264 specialists in 127 separate specialties.

### Views of Referring Doctors

The main users of the system are the doctors who refer to it. We asked two Iraqi physicians for their views on the system: EA is an obstetrician at Kirkuk, which is 300 km north of Baghdad, and HT is a surgeon in Erbil, 500 km north of Baghdad.

### Views of a Specialist

We analyzed neurology referrals because these referrals were mainly dealt with by a single specialist (VP) and there were previous publications on workload in other parts of the developing world [3]. We recorded the number and country of origin of the referrals, whether attachments were included, reply time, final diagnosis, number of email exchanges, and confidence in diagnosis.

## Results

**Figure 2:**
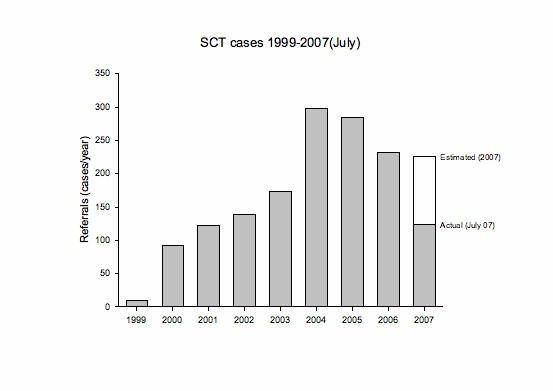
Annual referral rate to the Swinfen Charitable Trust (2007 figure was estimated from the actual number of cases received from January to July 2007)

From the inception of the service in July 1999 until June 2007, a total of 1466 referrals were received. The sustainability of the service is shown by the year-on-year referral pattern in Figure 2.

### Middle Eastern Referrals

As Table 1 shows, between July 2004 and June 2007, 283 referrals were received from the four Middle Eastern countries and 500 referrals from another 22 countries (the islands of St. Helena and Tristan da Cunha, while technically an Overseas Territory of the United Kingdom, were counted separately in view of their remoteness).

**Table 1:** Origin and numbers of cases referred from July 2004 to June 2007

**Middle East**		**Rest of World**	
Afghanistan	55	Bangladesh	123
Iraq	203	Bolivia	3
Kuwait	1	Cambodia	13
Pakistan	24	East Timor	23
		Ethiopia	11
		Gambia	2
		Lithuania	1
		Malawi	1
		Mozambique	7
		Nepal	129
		Papua New Guinea	36
		Russia	1
		Sierra Leone	1
		Solomon Islands	29
		Sri Lanka	23
		St. Helena (UK)	20
		Sudan	19
		Tibet	6
		Tristan da Cunha (UK)	21
		Uganda	3
		Uzbekistan	15
		Zambia	13
**Total**	**283**	**Total**	**500**

The 283 Middle Eastern cases resulted in 522 separate queries to specialists, 83 (16%) of which were unanswered. The median time to specialist reply for the queries was 24.3 hours (interquartile range 6.1-63.3).

Table 2 shows the breakdown by broad specialty group. The case mix from the Middle East was broadly similar to that from the rest of the world—the top three types of query were internal medicine, surgery, and pediatrics. However, the case mix was significantly different between the two regions with respect to certain specialities (*χ* ^2^_7_ = 84.2, *P* < .001), with more obstetric referrals and fewer referrals in medical specialties and radiology from the Middle East.

**Table 2:** Types of queries for the cases referred from July 2004 to June 2007

Specialty	Middle East	Rest of World
Allied health	0	8
Anesthetics	15	3
Emergency medicine	0	4
Internal medicine	149	275
Mental health	3	5
Nursing	3	4
Obstetrics and gynecology	93	43
Pediatrics	98	144
Pathology	17	25
Radiology	7	52
Surgery	128	208
Other	9	7
**Total**	**522**	**778**

### Views from Referring Doctors

This is the view of one of the Iraqi doctors who referred cases to us:

HT Telemedicine helped me in many situations and cases. In case number 1664, the opinions and advice from one of the plastic surgeons helped to elicit many other ideas in my mind to help the patient and do what satisfied him a lot. In case number 1682 with scalp avulsion, the advice given supported my opinion and I did the job more confidently. The results were excellent. In case number 1582, many opinions from surgeons and plastic surgeons helped me to save the life of the small girl who was injured in a terrorist attack in Mosul. This one was one of the most difficult cases I faced. The opinions from your Trust consultants have helped in some instances to change, and in others to support, my ways of treatment.

This is the view of another Iraqi doctor:

EA The tremendous usefulness and benefits of [the email referral system] to our...patients [in need] could be summarized in three points. The first benefit is its provision of modern clinical practice to our patients through the link with distinguished consultants in different specialties who have devoted their time and efforts to the help of patients freely and willingly. In this matter I can give an example of how one of our patients benefited from this link. A teenage girl presented with severe pulmonary edema due to pre-eclampsia at thirty weeks gestation. Upon our request, the advisory obstetrician and anesthetist responded swiftly, generously, and efficiently. Under their supervision, the local medical team managed to control the case and prolong pregnancy for another two weeks and managed to deliver her under spinal anesthesia. Happily, the mother and her premature baby left the hospital recovered. The second beneficial point is learning. The advisory consultant’s replies were not brief and concise messages, but they were long, detailed, informative, and instructive. They were rich in modern knowledge about the specific case. Occasionally, there were referrals to useful websites for further details. Our obstetric unit took advantage of this, and now most of our cesarean sections are being performed under spinal anesthesia instead of general due to the instruction of the advisory anesthetist. The last useful point, which is not perceptible, is its effects on the behavior and characters of the recipient doctors. The telemedical staff practised the virtues of love, care, and assistance to patients and doctors whom they do not know. This inspired us to behave similarly to our patients and to our colleagues.

### Views of a Specialist

A total of 26 email referrals were received by a single neurologist between July 2004 and June 2007 from Middle Eastern countries, 35% of all his email referrals during that period. Referrals were from Iraq (19), Afghanistan (5), Kuwait (1), and Pakistan (1). The median time to reply was 7 hours (range 0-7 days); 12 (44%) of the replies were sent on the same day, and 20 (77%) were sent within 2 days. The mean time to the last email was 5 days (range 0-27). Nine patients (35%) were ill enough to be in hospital and the rest were outpatients. Radiological images were attached to the referring email for 10 patients and clinical images, for eight. The neurologist requested a video clip for a further three patients. The neurologist was reasonably confident of the diagnosis in 20 (77%) patients. Most patients were dealt with by a single exchange of emails, but, for one patient, six exchanges were necessary. In all, 20 (77%) of the cases were regarded as being completed; in the remainder, information requested by the neurologist was not sent. There were 11 cases (42%) referred to other specialties, the most common being neuroradiology (4) and neurosurgery (3). The eventual diagnoses are shown in Textbox 1.

**Final diagnoses of neurology referrals (numbers of cases if more than 1)**

**Table:** 

cerebral tumours (4) tension headache (2) peripheral neuropathy (2) migraine bilateral optic neuritis central pontine myelinolysis cervical myelopathy cervical radiculopathy congenital muscular dystrophy dopa-responsive dystonia Erb’s palsy	hereditary hemorrhagic telangiectasia hereditary spastic paraplegia juvenile myoclonic epilepsy multiple sclerosis neurosarcoidosis nonstructural symptoms posttraumatic akinetic syndrome recurrent facial palsy spinal cord injury stroke

**Figure 3:**
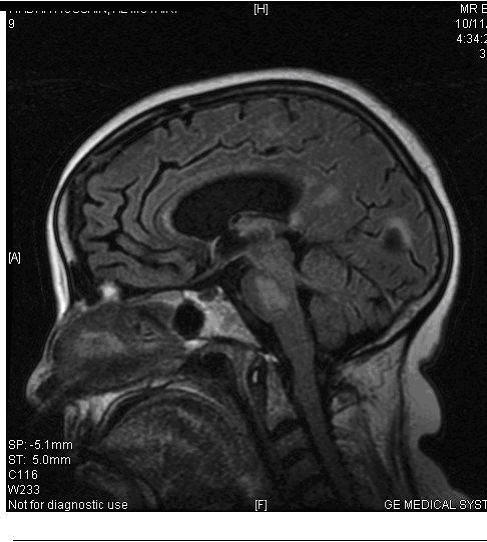
MRI scan of brain showing pontine and extrapontine myelinolysis

Six patients were referred for diagnosis, seven for management, and the rest for both diagnosis and management. In one patient, an exact diagnosis was not made because the patient died before further information was sent, but in the others, probable diagnoses were made and management plans suggested. For example, a patient in a persistent vegetative state was identified as having a locked-in syndrome due to central pontine myelinolysis (Figure 3), and appropriate rehabilitation was organized. A patient with unexplained coma and normal brain imaging was diagnosed as having neurosarcoidosis and the appropriate treatment was started. In both cases, the diagnosis was unsuspected by the referring physician and the management was radically changed.

## Discussion

We have demonstrated that a simple email system that connects doctors in the Middle East with specialists elsewhere in the world is feasible and sustainable over time, even in war-torn countries. The major difference between the Middle Eastern referrals and those from the rest of the world is the higher referral rate in obstetrics, which made up 18% of Middle Eastern referrals compared with 6% from the rest of the world. The views of the referring doctors about the service are positive. The neurology referrals are remarkably similar to those received in the industrialized world, and it was possible to make a confident diagnosis or management plan in 20 (77%) of the referrals handled by one neurologist.

Email telemedicine has not previously been used so extensively in obstetrics. The high proportion of obstetric and gynecology cases may have a number of causes. First, there have been obvious difficulties with clinical training over the last 10-20 years in the region in question. Second, in Iraq, the local community considers maternal death a disaster and an avoidable one and suspects negligence when it occurs, whereas death from medical or surgical illnesses is considered a decree of fate. That is why obstetricians attempt to provide the best care to their patients to avoid catastrophes, and one way of doing this is by asking the advice of the Swinfen Charitable Trust specialists. Third, the United Nations sanctions from 1990 until 2003, and the aftermath of the war in 2003, disabled the health care services in Iraq. That is why Iraqi doctors are now receiving patients in extreme conditions that have not been seen for many years, indeed often having to look for guidance in old textbooks. As well, women who have just given birth continue to convulse or bleed for hours at home because of the curfew from 10 pm till dawn each day, rather than being admitted to hospital straightaway. This is in addition to poor resources such as lack of even simple supplies and drugs. Finally, in Iraq, there is a high birth rate in the presence of very poor health care services and old-fashioned hospitals, the most modern being built and equipped in 1984. The urgency of the cases leaves the patients with no option but to accept these poor services, and, in turn, the doctors have no option other than to manage them even when they are to some extent beyond their knowledge and capability. That is why the advice from the Swinfen Charitable Trust specialists can be so valuable.

Despite the majority of Middle Eastern cases being referred from Iraq, relatively few were the direct result of conflict, and there were fewer trauma and fracture cases than from the rest of the world. There were also comparatively fewer referrals in infectious and tropical diseases. It is thought by the Trust’s administrators, who visited Iraq in 2004, that the relatively few trauma cases can be explained by the high level of local expertise developed during the conflict. The Director of Health, Basrah Governate, spoke ruefully of enduring 24 years of shelling and mortaring by Saddam Hussein’s armed forces from 1980 onward and said that the doctors and nurses had “learned in a hard school, how to manage with very little.” Similarly, local doctors are not likely to refer local infectious diseases, which may not even occur in the industrialized world.

We have described cases in which the email system made a significant difference to individual patients. This constitutes evidence of clinical effectiveness and is easy to show on a case-by-case basis. Unfortunately, it is not possible to comment on the magnitude of this effect—the overall percentage of people who benefited—because we have no follow-up data from the referring doctors. Out of consideration for the everyday difficulties, and sometimes the physical danger, of their medical practice, we did not request systematic information from them about the perceived benefits of each email consultation. For the same reasons, we do not have follow-up information that would enable us to comment on the safety of the system—the percentage of patients who have come to harm. Safety is a key component of the quality of any health care system, but it is impossible to measure when there is no follow-up or if there is no reliable gold-standard such as face-to-face examination by another specialist. This is difficult enough to achieve elsewhere in the developing world but is effectively impossible in a conflict situation. However, a small study of neurological patients in Bangladesh did show concordance between email and face-to-face management in four out of five patients [4].

The median reply time of 24 hours overall and 7 hours for the neurological consultations is probably faster than that for many referrals in UK hospitals and demonstrates the power of simple email as a method of communication. The time difference is such that most referrals sent in the morning in Iraq can be picked up in the United Kingdom before the start of the working day. There is naturally some slippage in the system, with 16% of queries being unanswered and therefore needing to be dealt with by another specialist; this is one cause of reallocation to other specialists, another being complex cases requiring the opinion of more than one specialist. The process does not raise ethical issues as it involves doctor-to-doctor consultations with the ultimate responsibility for patient treatment remaining with the referring doctor. We have not quantified the effect on medical education of the case-based learning mentioned by EA with possible beneficial effects extending well beyond the initial consultation.

The case mix of the neurological referrals is worth comment. Some were common and nonserious conditions, but others were life-threatening or unusual. All are typical conditions of the industrialized world and none are unique to the Middle East. The challenge for specialists is to tailor their advice to the local situation, which is often very different than their home-based practice. The shortage of neurological specialists throughout the developing world is more keenly felt in areas of conflict such as Iraq, where many specialists have left the country [5].

There have been other published examples of the use of telemedicine in the developing world. For example, consultations using radio transmission in rural Colombia [6] and Peru [7] have been reported, but these were between village health workers and a regional hospital rather than between doctors and specialists in a different country. Other systems operate between French-speaking Africa and Geneva, Switzerland [8] and between hospitals in the developing world and groups of specialists using a Web-based referral system [9], but these are primarily systems for medical education and telepathology, respectively, rather than for direct clinical use. The closest system to ours is that used in rural Cambodia [10,11], where mobile health care workers use email to communicate with specialists in Phnom Penh, Cambodia, and Boston, United States. Introduction of this system resulted in reduction of symptom duration at presentation compared with conventional care. There was a high level of patient satisfaction. Access to the Internet is a factor that limits uptake of this type of service, and this has recently been reviewed [12]. Almost all our cases and those from Cambodia were dealt with using access speeds of 56 kbit/s or less.

The work of the Swinfen Charitable Trust represents a drop in the ocean of health care delivery in the Middle East. However, because of the simplicity of the email telemedicine system and the relatively small associated cost, it can be easily expanded. There is no shortage of hospitals anxious to refer cases (and the Trust always welcomes new applications). Many specialists in the industrialized world are willing to give their time freely to help colleagues in less-developed parts of the world and find it easier to contribute their time by telemedicine than by travelling to deliver face-to-face care. The method provides an immediacy that most other improvements lack.

The great strength of email telemedicine is that it can improve access to health services among those most in need. However, its greatest weakness, at least in academic terms, is the lack of evidence supporting its clinical and cost advantages relative to traditional services, notwithstanding a number of retrospective reviews demonstrating the value to referrers [13-15]. This represents an important opportunity for research, and the Universitas 21 consortium of universities has recently formed an eHealth partnership with the Swinfen Charitable Trust [16] with a view to obtaining the follow-up necessary to make assessments of safety and effectiveness.
